# Frequency dependence 3.0: an attempt at codifying the evolutionary ecology perspective

**DOI:** 10.1007/s00285-015-0956-2

**Published:** 2016-02-01

**Authors:** Johan A. J. Metz, Stefan A. H. Geritz

**Affiliations:** Mathematical Institute and Institute of Biology, Leiden University, 2333 CA Leiden, The Netherlands; Department of Mathematics and Statistics, University of Helsinki, PO Box 68, 00014 Helsinki, Finland; Evolution and Ecology Program, International Institute for Applied Systems Analysis, 2361 Laxenburg, Austria; Naturalis Biodiversity Center, 2333 CR Leiden, The Netherlands

**Keywords:** Frequency dependence, Frequency independence, Weak frequency dependence, Invasion fitness, Meso-evolutionary statics, ESS theory, Feedback environment, Optimisation principle, Pessimisation principle, Adaptive dynamics, 92D15, 92D25, 92D40, 49K99

## Abstract

The fitness concept and perforce the definition of frequency independent fitnesses from population genetics is closely tied to discrete time population models with non-overlapping generations. Evolutionary ecologists generally focus on trait evolution through repeated mutant substitutions in populations with complicated life histories. This goes with using the per capita invasion speed of mutants as their fitness. In this paper we develop a concept of frequency independence that attempts to capture the practical use of the term by ecologists, which although inspired by population genetics rarely fits its strict definition. We propose to call the invasion fitnesses of an eco-evolutionary model frequency independent when the phenotypes can be ranked by competitive strength, measured by who can invade whom. This is equivalent to the absence of weak priority effects, protected dimorphisms and rock–scissor–paper configurations. Our concept differs from that of Heino et al. (TREE 13:367–370, 1998) in that it is based only on the signs of the invasion fitnesses, whereas Heino et al. based their definitions on the structure of the feedback environment, summarising the effect of all direct and indirect interactions between individuals on fitness. As it turns out, according to our new definition an eco-evolutionary model has frequency independent fitnesses if and only if the effect of the feedback environment on the fitness signs can be summarised by a single scalar with monotonic effect. This may be compared with Heino et al.’s concept of trivial frequency dependence defined by the environmental feedback influencing fitness, and not just its sign, in a scalar manner, without any monotonicity restriction. As it turns out, absence of the latter restriction leaves room for rock–scissor–paper configurations. Since in ‘realistic’ (as opposed to toy) models frequency independence is exceedingly rare, we also define a concept of weak frequency dependence, which can be interpreted intuitively as almost frequency independence, and analyse in which sense and to what extent the restrictions on the potential model outcomes of the frequency independent case stay intact for models with weak frequency dependence.

## Introduction

The concept of frequency dependence comes from population genetics. Textbooks on this subject are filled mainly with discrete time models without generation overlap and with fitnesses that depend only on the genotype and nothing else, determined by multiplying survival to adulthood with adult reproductive output. (In the case of biparental reproduction we shall measure the latter as the number of contributed alleles, i.e., half the number of offspring.^1^) Starting from this conveniently simple ecological scenario they immediately focus on gene frequencies, thereby hiding the unrealistic consequence that the population either grows to infinity or declines to zero (except for a few exceptional fitness configurations that make the long term population average of the fitnesses precisely equal to one). Within this ecologically simplified framework they concentrate on the change of gene frequencies for increasingly complicated genetic architectures. The first steps towards introducing a modicum of ecological realism were directly guided by the constraints of the chosen framework: let the relative fitnesses of the genotypes depend on the gene frequencies. Thus the concept of frequency dependence was born.

The only way to recover the textbook gene frequency equations in a discrete time model with non-overlapping generations with the population kept bounded by an environmental feedback loop, as in real populations, is to have the environment act on the fitnesses by multiplying them with a genotype independent term, so that this term cancels on dividing the densities of the genotypes by the total population density on the way to calculating gene frequencies. Such an environmental action may be thought of as the result of population increase leading to an indiscriminately killing environmental deterioration. When referring to population genetical models we shall henceforth assume that such a feedback loop is in place.

The introduction of more ecological realism, e.g., by considering the empirically ubiquitous cyclically driven size structured populations in continuous time kept in check by the delay in reaching reproductive size when food becomes scarce (e.g., De Roos and Persson 2013), torpedoes the simple fitness concept of population genetics, and therewith also the classical concept of frequency dependence. Yet the latter term is often felt to be heuristically useful in more general evolutionary discourse, and by now has gotten reified in the mind of the community, although in an unscientifically vague manner. The first authors attempting to remove this vagueness were Heino et al. (1998).^2^ In the meantime the conceptual tools for dealing with eco-evolutionary models have developed further to an extent that we feel compelled to come with an update.

With a few exceptions^3^ population geneticists have shown little interest in frequency dependence with evolutionary ecologists becoming the main users of the term. Their perspective differs in two main ways from that of population geneticists. The first difference is that evolutionary ecologists usually are interested in mechanistic life history detail, which therefore should be represented in the model. The second difference is that population geneticists focus on micro- and evolutionary ecologists on meso-evolutionary dynamics, more in particular meso-evolutionary statics, i.e., ESS theory.

*Terminology*  *Micro-evolution*: changes in gene frequencies on a population dynamical time-scale, the main realm of population genetics. *Meso-evolution*: the evolution of quantitative traits through repeated mutant substitutions, including the splitting of the evolutionary path into separate evolutionary lines, the main realm of quantitative genetics and adaptive dynamics. *Macro-evolution*: larger scale changes, like key innovations that can no longer be captured in terms of a fixed set of quantitative traits, or the effect of large scale environmental upheavals that irrevocably alter the selective arena. *ESS*:  “evolutionarily steady strategy”, that is, a strategy ($=$value of a trait vector) that, if sufficiently common, creates an environment such that no alternative strategy can invade. The abbreviation ESS actually derives from “evolutionarily stable strategy”. However, it was realised some time after the introduction of the concept that its definition still allows as ESSes strategies that are meso-evolutionarily repelling (Eshel 1983); hence our choice for a different interpretation of the abbreviation. *ESC*: evolutionarily steady coalition, that is, a combination of strategies that together create an environment such that no alternative strategy can invade. ESCs are the equilibria of meso-evolution (and perforce of macro-evolution, except that there there is a tendency for externally imposed environmental changes to interfere). When we use ESS in the adjectival form this is supposed also to comprise ESCs.

In general mechanistically-based eco-evolutionary models only allow the classical equations of population genetics to be extracted in the exceptional cases that a population model can be collapsed to the simple population model of the genetics textbooks, or when all phenotypes are closely similar so that selection is very weak. In the latter case the extracted equations for the, slow, genetic dynamics turn out to be those for small additive frequency independent fitness differences.

*Remark* We are referring here in both cases to the equations for the gene frequency dynamics. For the equilibria one can often get the textbook equations back, with the lifetime reproductive output of the different phenotypes as fitnesses (see e.g. Diekmann et al. 2003). However, if one writes out these fitnesses, taking account of the population structure and the environmental feedback loop (see Diekmann et al. 2003), one finds that these generically are frequency dependent, with the exception once again the unusual case where the feedback equally multiplicatively affects the phenotypic lifetime reproductive outputs.

For the weak selection dynamics case we have done the singular perturbation calculations, following Dercole and Rinaldi (2008) and Collet et al. (2013), only for the simplest possible cases. However, this should suffice for the point we want to make.

Beyond the case of weak selection the concept of frequency independence is in need of a proper definition, bringing it in line with ecological usage, which includes a far larger number of cases as frequency independent than allowed by the textbook definition that all fitness ratios should be constant.

For ESS theory it suffices to have a fitness concept that characterises the potential for population growth of mutant phenotypes in an environment set by resident phenotypes not yet influenced by the mutant. Thus the task becomes to introduce a concept of frequency (in)dependence for these invasion fitnesses that captures the intuitions that evolutionary ecologists usually attach to the term.

Although above we referred mainly to biparental reproduction, we shall below concentrate on clonal reproducers, to allow the ecological ideas to unfurl uncluttered by the complications of Mendelism, in line with common ecological practice, postponing a treatment of the latter complications to Sect. 7.

In Sects. 2 and 3 we review the minimal material needed to underpin our updated definition of frequency independence in Sect. 4. As the usefulness of a definition depends on how it performs, we review in Sect. 5 the main ideas of adaptive dynamics as the minimal formalised arena in which this performance may be tested. A concept is only worth its salt if any conclusions derived from its applicability show a certain degree of robustness. In Sect. 6 we formally introduce a kind of “almost frequency independence”, which we call weak frequency dependence, and explore the extent to which properties of frequency independent adaptive dynamics derived in Sect. 5 hold up under weak frequency dependence. The closing remarks in Sect. 8 discuss some of the methodological issues that inevitably go with the sort of concept engineering presented in this paper.

## Invasion fitness and fitness proxies: a short review

In the theory of structured populations anything outside an individual that influences its population dynamical behaviour, which by definition consists of impinging on the environment, giving birth and dying, is called *environment* (e.g., Metz and Diekmann 1986; Metz and Roos 1992; Metz et al. 1992; Metz 2008). Given this concept of environment we can always construct a Markovian representation of that behaviour in terms of a state space, transition probabilities that depend on the course of the environment and outputs that either deterministically depend on, or occur in a Poisson (or Poisson cluster) process with rates that depend on the individual’s state and the condition of the environment. Given the course of the environment, individuals independently move through their state spaces, the population state is a measure over this space, and the expectation of this measure, which is again a measure, moves according to a positive linear evolutionary system. The theory of such systems then tells that generally the expected size of a population in an ergodic environment will in the long run on average grow or decline exponentially (for details see Ferrière and Gatto 1995). The per capita rate of this growth $\rho$ is the sought after *fitness*.

The only environments that matter in ESS calculations are environments generated by a *resident* community. In nature populations are necessarily bounded. If this bound were too small the population would go extinct in too short a time for it to reach an ESS. Hence it is customary to assume that all populations are infinite in numbers although bounded in density, i.e., number of individuals per unit of area or volume. The community then follows a deterministic dynamics with as state space for each population a closed bounded subset of the cone of positive measures over the state space of the individuals, and as total state space the product of the state spaces of the comprising species, times the state spaces of the dynamics of any inanimate resources. With an infinitesimal amount of noise the states of such communities will, possibly after first losing some species, approach an “extinction preserving chain attractor” (Jacobs and Metz 2003; Gyllenberg et al. 2003). With larger amounts of noise the community will in general end up in a stochastic attractor, that is, a stationary distribution of community states (Jacobs and Schreiber 2006; Schreiber et al. 2011; Schreiber 2012; Roth and Schreiber 2014). We will throughout assume that the community attractor generates an ergodic environment (to all appearances exceptions to this assumption are rare), to be denoted as $E_{\mathrm {attr}}$. Moreover, in order to keep the argument simple, we shall assume that resident environments are uniquely determined by the traits of the residents (The arguments below straightforwardly extend to the general case where there can be more resident attractors generating different resident environments, but only at the cost of a good amount of verbal and notational clutter).

In locally sufficiently well mixed populations, so that the effect of the mutants on the environment is sufficiently diluted, a mutant population encounters a time function generated by the ergodic environmental process $E_{\mathrm {attr}}$ as its environmental input, which gets us the concept of *invasion fitness*. Mutants come singly. The theory of branching processes then tells us that a mutant with positive fitness has a positive probability to invade, while the probability to invade for a mutant with a non-positive fitness is zero (Remember, that for the residents we assume essentially infinite population sizes, so that a mutant population will not affect the environment before it has grown to a size where it can be treated deterministically).

The invasion fitness of a mutant population characterised by a trait value $Y \in {\mathcal X}$, ${\mathcal X}$ the set of possible trait values, or trait space, in a community with resident trait values $C=\{X_1 ,\ldots ,X_k \}$ thus equals

2.1 $$\begin{aligned} s(Y|C):=\rho (Y|E_{\mathrm {attr}} (C)). \end{aligned}$$

The use of | as separator of the arguments is borrowed from probability theory. Pronounce “the fitness of *Y* in a *C* background”, “the fitness of *Y* in the environment generated by *C*”, respectively. We shall refer to *s* as *fitness function*.

### *Assumption*

We prefer to leave the specification of ${\mathcal X}$ open as much as possible in view of the variety of trait spaces seen in nature. For the story in Sects. 3 and 4 it suffices that ${\mathcal X}$ is a separable metric space. The story in Sects. 5 and 6 proceeds as if ${\mathcal X}$ is a path connected subset of $\mathbb {R}^{k}$. However, we expect the developments in those sections to be at least extendable to finite unions of Banach manifolds. In all cases, we confine ourselves to “well-behaved” combinations of traits and ecologies: In Sects. 3 and 4 we assume that *s* is separately continuous in each of its two arguments, in Sects. 5 and 6 that it is $\hbox {C}^{2}$ in both arguments together.

Since resident populations do not go extinct or explode

2.2 $$\begin{aligned} s(X|C)=0\quad \hbox { for }X\in C. \end{aligned}$$

From the previous considerations it follows that for ESS calculations it suffices to know the sign of the invasion fitness. This suggests requiring that a definition of frequency dependence should depend only on properties of the fitness function and should respect sign equivalence.

Any quantity that is sign equivalent to invasion fitness is referred to as a fitness proxy. The iconic example, applicable in constant environments, is $$\ln (R_0 )$$, $$R_0 $$ the average lifetime offspring number. In the case of more than one birth state, $$R_0 $$ equals the dominant eigenvalue of the next generation operator, i.e., in the simplest case of finitely many birth states, a matrix $$\mathbf{B}$$ with as entries the average number of offspring born in state *i* over the lifetime of an individual born in state *j* (see e.g. Diekmann et al. 1990). Many ecologists refer to $$R_0 $$ just as fitness. The reasons for sticking to $$\rho $$ as the one and only fitness are that the definition of $$\rho $$ covers by far the widest range of ecological scenarios and that we feel that fundamental terms should have a unique meaning.

### *Remark*

By now the $$R_0 $$ concept has been extended to ever more complicatedly fluctuating environments such as periodic (Bacaër and Guernaoui 2006; Bacaër and Ait Dads 2012) or random ones (Bacaër and Khaladi 2013), and in abstracto without giving an effective calculation recipe by Thieme (2009) and Inaba (2012). However, where it is always easy to calculate $$\rho $$ at least numerically, for structured populations $$R_0 $$ is only easy to calculate for constant environments, and then usually far easier than $$\rho $$. [By the results of (Bacaër and Khaladi 2013), given a recipe for calculating the per capita population growth rate $$\rho $$, $$R_0 $$ can always be calculated indirectly from (with some slight abuse of notation) the equation $$\rho (R_0^{-1} \mathbf{R},\mathbf{S})=0$$, $$\mathbf{R}$$ the reproduction operator, $$\mathbf{S}$$ the survival operator. However, this only leads to a simple solution for unstructured populations, where $$\rho =\bar{{\beta }}-\bar{{\mu }}$$, $$\bar{{\beta }}$$ the time averaged birth rate, $$\bar{{\mu }}$$ the time averaged death rate: $$R_0 ={\bar{{\beta }}}/{\bar{{\mu }}}$$ (Bacaër and Guernaoui 2006).]

The frequency independent fitnesses of population genetics correspond to average lifetime offspring numbers in the constant virgin environment, $R_{0,\mathrm {V}}$. In these ecological scenarios the invasion fitness of a *Y* mutant heterozygote in a homozygous *X* resident population is

2.3 $$\begin{aligned} s(Y|X)=\ln \left( {R_0 (Y|X)} \right) =\ln \left( {R_{0,\mathrm {V}} (Y)/R_{0,\mathrm {V}} (X)} \right) . \end{aligned}$$

More generally, when the demographic parameters are at least $$\hbox {C}^{2}$$ in the invader trait, for small mutational steps $$R_0 $$ and $$\rho $$ relate as

2.4 $$\begin{aligned} \rho =\frac{\ln (R_0 )}{T_{\mathrm {b}} }+O\left( {\ln (R_0 )^{2}} \right) \end{aligned}$$

with $T_{\mathrm {b}}$ the average age at which residents of the type that spawned the mutant give birth (e.g. Durinx et al. 2008, Appendices 1, 2). This expression highlights that fitness and proxy may differ in dimension, in this case since $\rho$ is the relative rate of population increase in real time and $\ln (R_0 )$ the relative rate of population increase in (dimensionless) generation time.

For general models the dynamics of a mutant substitution often grossly matches that seen in the population genetical scenarios, with $s(Y|X)>0$ & $s(X|Y)>0$ the hallmark of a *protected dimorphism*, and in case of a substitution *s*(*Y*|*X*) the initial per capita growth rate of the mutant and *s*(*X*|*Y*) the final per capita decline rate of the former resident.

A further, partial, fitness proxy for the case of finitely many birth states is

2.5 $$\begin{aligned} Q:=-\det (\mathbf{I}-\mathbf{B}) \end{aligned}$$

(Metz and Leimar 2011). That is, $\ln (R_0 )>0$ if $Q>0$, and for continuous $\mathbf{B}$ and a path connected trait space, *C* is an ESC if $Q(Y|C)<0$ for all $Y\notin C$, and only if $Q(Y|C)\le 0$ for all $Y\notin C$. By the arguments in Appendix A of Rueffler et al. (2013) these properties also generically suffice for checking for frequency independence in the sense laid out in Sect. 4. However, it is not possible to conclude for a specific single *Y* from $Q(Y|C)<(=)\ 0$ that $\ln (R_0 (Y|C))<(=)\ 0$.

As final point we mention that the concept of invasion fitness extends considerably further than the case of locally well-mixed populations. All that matters is that mutant populations should early on go through a phase of (near) exponential growth, the relative rate of which we may then call invasion fitness. The arguments below apply whenever there exists a well-defined invasion fitness function *s*. This is, for example, also the case when individuals can be conceptually aggregated into meta-individuals (e.g., families or patches, see Metz 2013) forming a well-mixed population. This idea lies at the base of the meta-population invasion fitness proxy $$R_{\mathrm {m}} $$ introduced by Metz and Gyllenberg (2001) and Gyllenberg and Metz (2001) for the clonal case, and extended to the Mendelian case by Parvinen and Metz (2008), which on the level of the meta-individuals relates to invasion fitness as in well-mixed populations $$R_0 $$ does on the level of ordinary individuals.

Mendelian reproduction can be brought into the fully ecological fold by thinking of bodies as meta-individuals built of clonally reproducing genes (cf Metz 2013).

## On 1-dimensional environments and optimisation principles

The definitions of Heino et al. (1998) are based on the structure of the environmental feedback loop, where our updated ones will be based on the sign structure of the fitness function. The link between the two viewpoints is implicit in the material in three papers (Metz et al. 2008; Gyllenberg and Service 2011; Gyllenberg et al. 2011) dealing with the question under which conditions the ESSes of an eco-evolutionary model satisfy an optimisation principle. Below we give a quick summary of the relevant ideas from those papers.

### *Notation*

${\mathcal {C}}_n \subset \left\{ {\{X_1 ,\ldots ,X_n \}|X_i \in {\mathcal {X}}} \right\}$ will denote the set of possible trait coalitions with *n* members and ${\mathcal {C}}=\bigcup _{n=0}^\infty {{\mathcal {C}}_n }$ the set of all possible trait coalitions, i.e., sets of trait values that can coexist on a population dynamical time scale, with ${\mathcal {C}}_0$ having the virgin world as only member. The corresponding set of ecologically realisable environments will be denoted as $\mathcal {E}:=E_{\mathrm {attr}} ({\mathcal {C}})$.

*Terminology* A function $\psi :{\mathcal {C}}\rightarrow \mathbb {R}$ such that for any constraint on ${\mathcal {X}}$ the ESCs can be calculated by maximising $\psi$, is called an *optimisation principle*. We refer to the existence of such a principle for an eco-evolutionary model as the model having an optimisation principle.

### **Lemma 3.1**

(Gyllenberg et al. 2011) If $\psi$ is an optimisation principle and $C \in \mathcal {C}$ then

3.1 $$\begin{aligned} \psi (X)=\psi (C)\quad \hbox {for all}\quad X\in C. \end{aligned}$$

### **Lemma 3.2**

(Metz et al. 2008) If $\psi$ is an optimisation principle, then a mutant *Y* can invade in *C*, and then also will take over, if $\psi (Y)>\psi (C)$, and cannot invade when $\psi (Y)<\psi (C)$.

### **Definition**

We say that the environment

${\bullet }$*acts 1-dimensionally*, or that *the environmental feedback loop is 1-dimensional* iff there exist functions $\varphi :{\mathcal {E}}\rightarrow \mathbb {R}$ and $\beta :{\mathcal {X}}\times \mathbb {R}\rightarrow \mathbb {R}$ such that $\rho (X|E)=\beta \left( {X,\varphi (E)} \right)$,

${\bullet }$*acts monotonically 1-dimensionally*, or that *the environmental feedback loop is monotonic 1-dimensional* iff the functions ${\varPhi }$ and $\beta$ can be chosen such that $\beta$ increases in its second argument.

${\bullet }$*acts effectively 1-dimensionally*, or that *the environmental feedback loop is effectively 1-dimensional* iff there exist functions $\varphi :{\mathcal {E}}\rightarrow \mathbb {R}$ and $\beta :{\mathcal {X}}\times \mathbb {R}\rightarrow \mathbb {R}$, such that $\mathrm{sign}\left( {\rho (X|E)} \right) =\mathrm{sign}\left( {\beta \left( {X,\varphi (E)} \right) } \right)$,

${\bullet }$*acts effectively monotonically 1-dimensionally*, or that *the environmental feedback loop is effectively monotonic 1-dimensional* iff the functions ${\varPhi }$ and $\beta$ can be chosen such that $\beta$ increases in its second argument.

A similar set of concepts can be defined for the traits. We only give the most relevant one.

### **Definition**

We say that the traits *act effectively monotonically 1-dimensionally* iff there exist functions $\psi :{\mathcal {X}}\rightarrow \mathbb {R}$ and $\alpha :\mathbb {R}\times {\mathcal {E}}\rightarrow \mathbb {R}$, increasing in its first argument, such that $\mathrm{sign}\left( {\rho (X|E)} \right) =\mathrm{sign}\left( {\alpha \left( {\psi (X),E} \right) } \right)$.

Note that the functions $\psi$ and ${\varPhi }$ are only determined up to monotonic transformations.

The interesting point is that effective monotonic 1-dimensional action of the environment and of the traits are linked:

### **Theorem 3.3**

(Metz et al. 2008) Effective monotonic 1-dimensional action of the environment and effective monotonic 1-dimensional action of the trait imply each other. Not only that, if the trait or environment act effectively monotonically 1-dimensionally, it is possible, by choosing $\psi$ or ${\varPhi }$ such that $\psi (X)=-\varphi \left( {E_{\mathrm {attr}} (X)} \right)$, to write

3.2 $$\begin{aligned} \mathrm{sign}\left( {s(Y|X)} \right)= & {} \mathrm{sign}\left( {\psi (Y)+\varphi \left( {E_{\mathrm {attr}} (X)} \right) } \right) \nonumber \\= & {} \mathrm{sign}\left( {\varphi \left( {E_{\mathrm {attr}} (X)} \right) -\varphi \left( {E_{\mathrm {attr}} (Y)} \right) } \right) \nonumber \\= & {} \mathrm{sign}\left( {\psi (Y)-\psi (X)} \right) \end{aligned}$$

In addition, an eco-evolutionary model has an optimisation principle iff the environment or the trait act effectively monotonically 1-dimensionally.

Metz et al. (2008) dubbed functions $\varphi :\mathcal {E}\rightarrow \mathbb {R}$ with the property that it is possible to calculate ESSes by minimising $\varphi \left( {E_{\mathrm {attr}} (X)} \right)$ pessimisation principles. Where $\psi$ can be thought of as measuring some sort of absolute quality of the phenotypes, ${\varPhi }$ can be thought of as measuring the quality of the environment: a decrease of ${\varPhi }$ means that less phenotypes can increase in numbers in the new environment. It follows from (3.2) that each mutant substitution increases $\psi (X)$ and decreases $\varphi \left( {E_{\mathrm {attr}} (X)} \right)$, so that in the end only the best possible types survive in the worst possible environment.

The following obvious corollary is the essential ingredient behind the ideas in Sect. 6.

### **Corollary 3.4**

(new) An eco-evolutionary model has an optimisation principle (or a monotonically 1-dimensionally acting environment, or monotonically 1-dimensionally acting traits) iff there exist functions $\psi :{\mathcal {X}}\rightarrow \mathbb {R}$ and $f:{\mathcal {X}}\times {\mathcal {X}}\rightarrow \mathbb {R}^{+}$ such that it is possible to write the invasion fitness as

3.3 $$\begin{aligned} s(Y|X)=f(Y,X)\left( {\psi (Y)-\psi (X)} \right) . \end{aligned}$$

For given continuous *s* and $\psi$ the requirement that *f* is also continuous determines *f* uniquely.

It is also possible to write down criteria for the existence of an optimisation principle in terms of no more than the sign structure of the fitness function:

### **Theorem 3.5**

(Gyllenberg and Service 2011) An eco-evolutionary model has an optimisation principle iff

3.4 $$\begin{aligned} (\mathrm{i})\ s(Y|X)<0\Leftrightarrow s(X|Y)>0 \end{aligned}$$

(*sign anti-symmetry*) and

3.5 $$ \begin{aligned} (\mathrm{ii})\ s(Y|X)\ge 0\ \& \ s(Z|Y)\ge 0\Rightarrow s(Z|Y)\ge 0 \end{aligned}$$

(*sign transitivity*).

The following lemmas give a more ecological slant.

### **Lemma 3.6**

(new) Condition (i) is equivalent with:

(i’a) there are no pairs of traits values (*Y*, *X*) such that

3.6 $$ \begin{aligned} s(Y|X)\le 0\ \& \ s(X|Y)<0 \end{aligned}$$

(absence of *weak priority effects*) and

(i’b) there are no pairs of traits values (*Y*, *X*) such that

3.7 $$ \begin{aligned} s(Y|X)\ge 0\ \& \ s(X|Y)>0 \end{aligned}$$

(absence of *weak protected polymorphisms*).

### **Lemma 3.7**

(Gyllenberg and Service 2011) Given (i), condition (ii) is equivalent with

(ii’) there do not exist triples such that

3.8 $$\begin{aligned} s(Y|X)\ge 0,\quad s(Z|Y)\ge 0,\quad s(X|Z)>0 \end{aligned}$$

(absence of *weak rock-scissor-paper configurations*).

## Frequency dependence 3.0: towards an improved definition

We start with a résumé of Heino et al. (1998). These authors distinguish four types of frequency dependence: none (below to be referred to as classical frequency independence or as just classical), trivial, weak and strong, with classical subsumed under trivial, and strong subsumed under weak. Classical frequency independence refers to the case where the environmental feedback works through indiscriminate killing (or, in the case of non-overlapping generations any multiplicative reduction of the average offspring number). In that case it is possible to describe the feedback in terms of a single variable, the additional killing rate (in continuous time, ratio in discrete time). As an extension from this they introduce the term trivial frequency dependence for all the cases where the environment acts 1-dimensionally. Weak frequency dependence refers to the cases where the environmental feedback loop needs more variables for its description. One of the properties of trivial frequency dependence is that it generically prevents polymorphisms by the extended competitive exclusion principle (Levin 1970; Meszéna et al. 2006). Finally strong frequency dependence is taken to refer to the cases where a higher dimensional feedback loop imparts an advantage to rareness.

Our proposed new definitions differ from those of Heino et al. (1998) in two respects. First of all, we base our definitions entirely on the signs of the invasion fitnesseses. Secondly, we focus on eco-evolutionary models, whereas Heino et al. implicitly considered only ecological models. The difference is that for eco-evolutionary models we always consider the full ${\mathcal {C}}$ in one go, whereas for ecological models the focus is on one trait-*n*-tuple at a time.

### *Remark*

Here is one more reason why it is better to focus on the sign of invasion fitness rather than on fitness itself. An environmental feedback loop that acts by just adding a factor to $\rho$ (the hallmark of classical real time frequency independence) generally does not do so with $\ln (R_0 )$. Conversely, a feedback loop that acts by just adding a factor to $\ln (R_0 )$ (the hallmark of classical frequency independence in generation time) generally does not do so with $\rho$. Similarly, an environment that acts 1-dimensionally in determining $\rho$, generally does not do so in determining $\ln (R_0 )$, and an environment that acts 1-dimensionally in determining $\ln (R_0 )$, generally does not do so in determining $\rho$ (In all cases the ecological scenario from population genetics is the main exception). However, the dimension of the environmental action is the same when it comes to determining $\mathrm{sign}(\rho )$, $\mathrm{sign}\left( {\ln (R_0 )} \right)$, or the sign of any other fitness proxy. And where the frequency dependence concepts of Heino et al. (1998) depend on a narrow focus on $\rho$, our updated concepts are independent of whether we consider invasion fitness proper, $\ln (R_0 )$, or whatever other fitness proxy.

One-dimensionality of the feedback loop generically implies sign anti-symmetry of the invasion fitnesses, that is, of two trait values that do not happen to be an exact competitive match, one is either irrevocably better of irrevocably worse than the other (The proof of this statement in Appendix 1 admittedly makes some simplifying assumptions. However, this should suffice for the philosophical point we want to make here). As sign anti-symmetry seems sort of the hallmark of the ecologist’s intuition about frequency independence (below we shall see that there is more) we propose to take this property as a main criterion instead of 1-dimensionality of the feedback. [Unlike Heino et al. (1998) we thus no longer distinguish between frequency independence and trivial frequency dependence.]

What is still missing in requiring sign anti-symmetry, is that only pairwise comparisons of strategies are considered. Doing so leaves open the possibility of rock-scissor-paper cycles, the presence of which in recent years has come to be seen as yet another prominent manifestation of frequency dependence. Hence we add the requirement that there are no such cycles as a second criterion to our definition.

By the results of Gyllenberg and Service (2011), under conditions of sign anti-symmetry the absence of rock-scissor-paper cycles is equivalent to sign transitivity of the fitness function. The results in (Gyllenberg and Service 2011; Gyllenberg et al. 2011) reviewed above then show that after introducing the conditions of sign anti-symmetry and sign transitivity there is no need for further conditions, since these conditions together imply that one can linearly order the phenotypes (or rather equivalence classes of mutually neutral phenotypes) according to their “evolutionary quality”, which is also the main qualitative property of the frequency independent fitnesses of the population geneticists.

We summarise the above considerations in the following

### **Definition**

We shall call an eco-evolutionary model frequency independent iff its invasion fitness function is both sign anti-symmetric and sign transitive.

The existence of a linear order on the phenotypes is in turn equivalent to the existence of an optimisation principle sensu Metz et al. (2008), which is nothing but a numerical representation of this order. Taking sign transitivity on board in addition to sign anti-symmetry as defining frequency independence thus makes the latter equivalent to the existence of an optimisation principle. This in turn was proven by Metz et al. (2008) to be equivalent to the existence of a similar ordering of the environments, in that one environment is better than another if any type that can just survive in the former will perish in the latter and any type that just survives in the latter, will flourish in the former. For symmetry reasons a numerical representation of the latter order has been dubbed a pessimisation principle.

It may seem that with the introduction of pessimisation principles we are back at the 1-dimensional environmental action of Heino et al. (1998). However, our updated concept of frequency independence is weaker in the sense that the 1-dimensionality is only up to sign equivalence, as well as stronger in the sense that the dependence of the sign of the invasion fitness on the environment through a pessimisation principle is necessarily monotonic, just like the dependence on the traits through an optimisation principle. Not requiring the 1-dimensional environmental action of the environment to be monotonic opens up the possibility for rock-scissor-paper cycles.

Finally, we propose to keep the term strong frequency dependence as it appears useful in ecological discourse. However, where the terms frequency independence and frequency dependence can be applied to the families of ecological models that form the basis of eco-evolutionary models, the term strong frequency dependence can only usefully be applied to single ecological models, as in eco-evolutionary models there generically are trait values that can be replaced by other trait values in a trait substitution, in which case there is no strong frequency dependence, even in models where there exist also pairs of trait values that can occur in protected dimorphisms and thus by definition are subject to strong frequency dependence.

## A formalised arena for testing the new concept: adaptive dynamics

A second reason to do away with Heino et al.’s term weak frequency dependence is that we have another use for the term that we find more useful as well as natural. Basically we want to refer to an eco-evolutionary model that is almost frequency independent as weakly frequency dependent. However, before giving a definition in Sect. 6 we should explain the applications that we envision, as these determine the precise form of that definition: we want to use the concept as tool in adaptive dynamics arguments.

*Adaptive dynamics* (AD) is a mathematical framework for dealing with meso-evolutionary dynamics in ’realistic’ ecological settings. It has ESS theory for its statics. The realism at the ecological end is bought by making strong simplifying assumptions at the population genetical end (Metz et al. 1996; Dieckmann and Law 1996; Geritz et al. 1998). The two main ones are (i) separation of the population dynamical and mutational timescales and (ii) clonal inheritance. Two more assumptions are made to arrive at an effective toolbox, (iii) smooth (at least $\hbox {C}^{2})$ fitness functions, and (iv) sufficiently small mutational steps (Fortunately, under assumptions (iii) and (iv) assumptions (i) and (ii) can be relaxed quite a bit, e.g., Meszéna et al. 2005; Metz and Kovel 2013). Away from community dynamical bifurcation points and the evolutionarily singular points to be discussed below, for a sufficiently small mutational step a mutant’s invasion irrevocably leads to its substitution (this has so far been proved only for ODE community models (Geritz 2005; Dercole and Rinaldi 2008; Collet et al. 2013) but the structure of the proof suggests that the result has a far wider applicability).

Adaptive dynamics has two workhorses, its *canonical equation* (CE), and so-called *Pairwise Invasibility Plots* (PIPs). The CE is a differential equation describing the movement of the trait vector over meso-evolutionary time based on two subsequent limits, (1) from a full individual based population model to so-called trait substitution sequences where one only considers how the trait vectors change stepwise as a result of mutants invading and becoming the new resident, followed by (2) a limit where the mutational steps go to zero, where in both cases time is rescaled to keep the trait evolution in view [Dieckmann and Law 1996; Champagnat 2003; Durinx et al. 2008; Méléard and Tran 2009; Champagnat and Méléard 2011; Collet et al. 2013; Metz and Kovel 2013; Metz and Jansen in preparation)]. Below we give its form for Mendelian diploids; for haploids or clonal reproducers the factor 2 has to be removed.

5.1 $$\begin{aligned} \frac{\hbox {d}X}{\hbox {d}t}=2N_{\mathrm {e}} \theta \varvec{{\Sigma }}\ G(X), \end{aligned}$$

where $N_{\mathrm {e}}$ is the effective population size (as in the population genetics textbooks), $\theta$ the mutation probability per birth event, ${\Sigma }$ the covariance matrix of the mutational steps, and $G(X):=\partial _1^{\mathrm {T}} s(X|X)$ the so-called *selection gradient*.

### *Notation*

When applied to a function with a *k*-dimensional vector-argument $\partial$ stands for $\left( {\partial /{\partial x_1 },\ldots ,\partial /{\partial x_k }} \right)$, while $\partial _i$ refers to the application of $\partial$ to the *i*-th argument of a function specified as having two or more vector-arguments.

*Terminology* Points $X^{*}$ at which $G(X^{*})=0$ are called *evolutionarily singular strategies* (*ess-es*).

Near ess-es various other phenomena occur, resulting i.a. in the convergence towards the CE becoming slower and slower the closer the trait substitution sequence approaches an ess.

### *Convention*

When writing about the CE we always assume that the *X* that we consider are further than $\delta$ from an ess. The correspondingly modified adaptive state space we shall refer to as ${\mathcal {C}}_1^{\backslash \delta }$.

### *Notation*

$\mathbf{C}<(\le ,\ge ,>)\ 0$ means that $X^{T}\mathbf{C}X<(\le ,\ge ,>)\, 0$ for all $X\ne \mathbf{0}$.

Thanks to the fact that $s(X|X)=0$, the invasion fitness function can locally around the diagonal be expanded as

5.2 $$\begin{aligned} s(X+V|X+U)= & {} B(V-U)+V^{T}{} \mathbf{C}_{00} V+2V^{T}{} \mathbf{C}_{01} U+U^{T}{} \mathbf{C}_{11} U+\hbox {h.o.t.} \nonumber \\= & {} \left[ {B-\left( {V+U} \right) ^{T}{} \mathbf{C}_{11} -2V^{T}{} \mathbf{C}_{01} } \right] \left( {V-U} \right) +\hbox {h.o.t. }\nonumber \\= & {} \left[ {B+\left( {V+U} \right) ^{T}{} \mathbf{C}_{00} +2U^{T}\mathbf{C}_{10} } \right] \left( {V-U} \right) +\hbox {h.o.t.} \end{aligned}$$

with $B:=\partial _1 s(X|X)=-\partial _2 s(X|X)$, $\mathbf{C}_{ij} :=\frac{1}{2}\partial _i^T \partial _j s(X|X)$, $\mathbf{C}_{00} +\mathbf{C}_{01} +\mathbf{C}_{10} +\mathbf{C}_{11} =0$, $\mathbf{C}_{10} =\mathbf{C}_{01}^T$, all dependent on *X*. Hence at ess-es

5.3 $$\begin{aligned} s(X^{*}+V|X^{*}+U)=V^{T}{} \mathbf{C}_{00} V+2V^{T}{} \mathbf{C}_{01} U+U^{T}{} \mathbf{C}_{11} U+\hbox {h.o.t.} \end{aligned}$$

*Terminology* A strategy that is uninvadable by nearby strategies is called a *local ESS*. In ESS theory $2\mathbf{C}_{00}$ is usually called the *selection Hessian*, and then denoted as $\mathbf{H}$.

ESSes are special kinds of ess-es. An ess is a local ESS if $\mathbf{H}(X^{*})<0$, and only if $\mathbf{H}(X^{*})\le 0$.

*Terminology* An ess is called *strongly attractive* if for any non-singular mutational covariance matrix it is robustly^4^ approached from nearby by the solution of the CE.

### **Theorem 5.1**

(Leimar 2009) An ess is strongly attractive iff

5.4 $$\begin{aligned} \mathbf{C}_{11} (X^{*})-\mathbf{C}_{00} (X^{*})>0. \end{aligned}$$

**Fig. 1:**
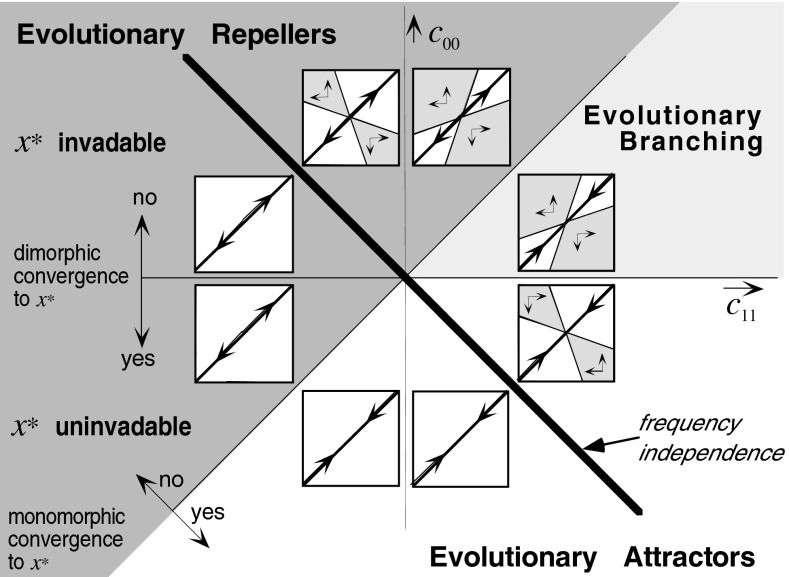
The different types of ess-es for 1-dimensional trait spaces. In each of the small diagrams the dimorphic coexistence region is indicated in *grey*, while the diagonal represents the monomorphic states. The *arrows* give the directions in which mutants with small effect can take over

One-dimensional trait spaces, thanks to their linear ordering, come with stronger tools, the main one of which are *PIP*s (Matsuda 1985; Tienderen and Jong 1986; Metz et al. 1996; Geritz et al. 1998). PIPs are sign plots of the fitness function. Generically the zero-set of the fitness function consists of one or more smooth curves, including the diagonal, which divide a PIP into regions, with contiguous regions bearing opposite sign. The ess-es occur where another zero-curve crosses the diagonal. PIPs can be used to analyse the qualitative behaviour of trait substitution sequences in a similar manner as one uses cobwebbing to analyse recurrences in one variable, although with a twist. Not only are the steps stochastic, determined by the mutation process and the probability that a mutant invades, but near some types of ess-es the trait substitution sequence can step from ${\mathcal {C}}_1$ to ${\mathcal {C}}_2$, and in a subset of cases also in the opposite direction. To see this, imagine flipping the PIP over its diagonal and putting the result on top of the original. In the $++$ regions two phenotypes will coexist through strong frequency dependence. For the local analysis one uses that around the crossing the second zero curve can be written as $(y-y^{*})=\left( {{c_{11} }/{c_{00} }} \right) (x-x^{*})$. See Fig. 1, and for further details (Metz et al. 1996; Geritz et al. 1998, 1999).

The CE naturally extends to the polymorphic components of ${\mathcal {C}}$, and one can there also fall back on PIP arguments by mentally forbidding all but one phenotype to mutate.

*Terminology* A zero of $(G_1 (C),\ldots ,G_n (C))$, $G_i :=\partial _1^T s(X_i |\{X_1 ,\ldots ,X_n \})$, is called an *evolutionary singular coalition* (esc), denoted as $C^{*}$. A strategy coalition that cannot be invaded by strategies that are close to any of its members is called a *local ESC*.

ESCs are special kinds of esc-s. An esc is a local ESC if all selection Hessians $\mathbf{H}_i (C^{*})<0$, $\mathbf{H}_i (\{X_1 ,\ldots ,X_n \}):=\partial _1^T \partial _1 s(X_i |\{X_1 ,\ldots ,X_n \})$, and only if all $\mathbf{H}_i (C^{*})\le 0$.

For frequency independent fitness functions having $\psi$ for an optimisation principle, $-\psi$ acts as Lyapunov function. Hence a trait substitution sequence converges to a maximum of $\psi$. This may be a local maximum when the size of the mutational steps are bound to be small, and will be a global maximum when there are no such restrictions (i.e., the support of the distribution of mutational steps coincides with ${\mathcal {X}})$. A result for the CE, derived in Appendix 2, is

5.5 $$\begin{aligned} \frac{G(X)}{\left\| {G(X)} \right\| }=\frac{\partial ^{T}\psi }{\left\| {\partial ^{T}\psi } \right\| }, \end{aligned}$$

that is, the selection gradient everywhere points in the same direction as the gradient of $\psi$. From (5.5) we can thus once more conclude that also for the CE $\psi$ increases over evolutionary time (see Appendix 2). Furthermore, at ess-es

5.6 $$\begin{aligned} \mathbf{C}_{11} =-\mathbf{C}_{00}\quad \hbox { and }\quad \mathbf{C}_{01} =\mathbf{C}_{10} =0 \end{aligned}$$

(Appendix 2), as local expression of the sign anti-symmetry of *s*. From (5.6) it follows that at any ESSes the criterion for strong convergence $\mathbf{C}_{11} -\mathbf{C}_{00} >0$ holds true. For 1-dimensional trait spaces the restrictions (3.4) and (3.5) make for PIPs with very special properties (see Metz et al. 2008, Fig. 2), one aspect of which is that they are skew symmetric (corresponding to the sign anti-symmetry of *s*). In Fig. 1 this amounts to the frequency independent case being located on the fatly drawn anti-diagonal.

## Weak frequency dependence 2.0

### **Definition**

We shall call a fitness function *sweakly frequency dependent* if there exist a $\hbox {C}^{2}$ function $\psi : {\mathcal {X}}\rightarrow \mathbb {R}$ and a $\hbox {C}^{2}$ function $f:{\mathcal {X}}\times {\mathcal {X}}\rightarrow \mathbb {R}^{+}$ such that *s* is $\hbox {C}^{2}$-close to the frequency independent fitness function $\tilde{s}:(Y,X)\mapsto \tilde{s}(Y|X)= \quad f(Y,X)\left( {\psi (Y)-\psi (X)} \right)$.

The reason for opting for $\hbox {C}^{2}$-closeness is that $\hbox {C}^{0}$-closeness guarantees the similarity of the sets $\{(Y,X);s(Y|X)\ge 0\}$ and $\{(Y,X);\psi (Y)\ge \psi (X)\}$, so that any PIPs derived from *s* from a distance will look similar to those of a frequency independent fitness function. $\hbox {C}^{0}$-closeness of the first derivatives guarantees that the canonical equation connected to *s* behaves similar to a CE going with a frequency independent fitness function. $\hbox {C}^{0}$-closeness of the second derivatives guarantees that close to singular points the geometry of *s* is similar to that of a frequency independent fitness function. These ideas are made more precise in the following lemma.

*Terminology* Below we shall for a weakly frequency dependent fitness function *s* with frequency independent counterpart $\tilde{s}$ refer to $\left\| {s-\tilde{s}} \right\| _{C^{2}}$ as $\varepsilon$, and to $\psi$ as *associated approximate optimisation principle*.

An optimisation principle $\psi$ will be called *regular* when (i) it is $\hbox {C}^{2}$, (ii) it has finitely many singular points, i.e., solutions of $\partial \psi (X)=0$, (iii) its values at its singular points are all different, (iv) its Hessians at those singular points are non-singular.

### **Lemma 6.1**

For weakly frequency dependent fitness functions with an associated approximate optimisation principle $\psi$

6.1 $$\begin{aligned} (\mathrm{i})\ D(\{(Y,X);s(Y|X)\ge 0\},\{(Y,X);\psi (Y)\ge \psi (X)\})=\hbox {O}(\varepsilon ), \end{aligned}$$

where $D(A,B)=\max \{\mathop {\sup }\limits _{Y\in B} \mathop {\inf }\limits _{X\in A} d(X,Y),\mathop {\sup }\limits _{X\in A} \mathop {\inf }\limits _{Y\in B} d(X,Y)\}$, $A,B\subset {\mathcal {X}}$, and *d* the distance in ${\mathcal {X}}$,

(ii) for all $\delta$ and all $\{X\}\in C_1^{\backslash \delta }$

6.2 $$\begin{aligned} \left\| {\frac{G(X)}{\left\| {G(X)} \right\| }-\frac{\partial ^{T}\psi }{\left\| {\partial ^{T}\psi } \right\| }} \right\| =\hbox {O}(\varepsilon ) \end{aligned}$$

(iii) at ess-es

6.3 $$\begin{aligned} \left\| {\mathbf{C}_{00} +\mathbf{C}_{11} } \right\| =\hbox {O}(\varepsilon )\quad \hbox { and }\quad \left\| {\mathbf{C}_{01} } \right\| =\hbox {O}(\varepsilon ). \end{aligned}$$

(i) means that a weakly frequency dependent fitness function is close to sign anti-symmetric in the large, (iii) that it is close to sign anti-symmetric also locally around any ess-es. This specialises to PIPs in that PIPs are close to skew symmetric in the large as well as close to skew symmetric locally around ess-es. Finally, (ii) and (iii) together mean that for a regular mutational covariance matrix and sufficiently weak frequency dependence with a regular approximate optimisation principle the flow from the CE moves the trait vector to a strongly locally attracting ESS.

### **Lemma 6.2**

If a $\psi$ associated with a weakly frequency dependent fitness function is regular, then for sufficiently small $\varepsilon$ its local ESSes (which lie near the maxima of $\psi$) coincide with the monomorphically attracting ess-es; moreover, their attractivity is strong.

In contrast to the fully frequency independent case, a weakly frequency dependent fitness function may engender strong frequency dependence, although only in a very small part of $\bigcup _n {\left\{ {\{X_1 ,\ldots ,X_n \}|X_i \in {\mathcal {X}}} \right\} }$. This can be seen in Fig. 1. Moving slightly upward from (to the right of) the fatly drawn anti-diagonal brings one from the frequency independent cases on the anti-diagonal to weakly frequency dependent cases that exhibit a narrow cone of protected dimorphisms.

From Fig. 1 it can also be read of that for 1-dimensional trait spaces near the monomorphically attracting ESSes any protected dimorphism contracts through subsequent mutational steps (the diagrams in the lower right region), while those near monomorphically repelling ess-es expand (the diagrams in the upper left region); in the first case the dimorphisms are almost surely again after a while replaced by monomorphisms through a mutant substitution to outside the coexistence region, in the second case a similar substitution happens with high probability (i.e., a probability that gets closer to one the closer *s* gets to $\tilde{s}$). The situation in the large is captured by the following lemma (proved in Appendix 3).

### **Lemma 6.3**

If a $\psi$ associated with a weakly frequency dependent fitness function is regular, then for sufficiently small $\varepsilon$ the protected polymorphisms do not host esc-s, and perforce no ESC-s.

### **Corollary 6.4**

If a $\psi$ associated with a weakly frequency dependent fitness function is regular, then for sufficiently small $\varepsilon$ a trait substitution sequence starting from a polymorphic initial condition will almost surely after a while step to the monomorphisms.

## Mendelian diploids

From an extreme ecological perspective, with bodies seen as meta-individuals built of clonally reproducing genes, heterozygote superiority causes strong frequency dependence. However, population geneticists, who traditionally start from bodies as primary entities rather than genes, commonly ascribe fitnesses to the phenotypes of bodies. As a result their view on frequency independence is that the ratios of those phenotypic fitnesses should be constant, while their view on frequency dependence is that those fitness ratios depend on the relative allele frequencies, and nothing else. However, the population geneticists’ fitness concept, and with this any derived concepts, does not extend beyond the special ecological scenarios that form the standard population geneticists’ playground, with the different life phases both neatly separated and synchronised.

When looking at more general ecological scenarios we run into the problem that it is not immediately clear how the definition of invasion fitness given in Sect. 2 should be extended to Mendelian populations. For monomorphic resident populations there is no problem as a mutant allele enters the population in only a single phenotypic guise, that of the heterozygote with its resident counterpart, and thus behaves just like a clonal reproducer. However, the situation for polymorphic resident populations is different. There we have to let the phenotype associated with the invading mutant consist of a list of the trait vectors that the mutant allele produces in all the different genetic backgrounds in which it may find itself (for worked examples see Metz 2008; Metz and Leimar 2011). Unfortunately, this multiplication of trait vectors screws up the straightforward reasoning of Sects. 3–5.

The next step then should be to see what can and cannot be rescued from our arguments for clonal populations. One strategy is to confine ourselves to populations that stay monomorphic. As it happens, rephrasing (3.4) for Mendelian populations also assumes away the possibility that somewhere along the line heterozygote superiority raises its ugly head. If we generically denote the map from genotype to phenotype with $${\varPhi }$$ (so $${\varPhi }$$ may have different arguments dependent on the genetic scenario we envisage) and alleles as $$A_i $$, $$B_i $$ or $$C_i $$, the immediate counterpart of (3.4) becomes

7.1 $$\begin{aligned} s({\varPhi } (A_1 A_2 )|{\varPhi } (A_1 A_1 ))>0\Leftrightarrow s({\varPhi } (A_1 A_2 )|{\varPhi } (A_2 A_2 ))<0. \end{aligned}$$

However, since Lemma 3.1 is predicated on clonal reproduction we have to assume in addition that the ecology does not allow unprotected genetic dimorphisms. Finally, we replace (3.8) by the assumption that there exist no triples $(A_1 ,A_2 ,A_3 )$ such that

7.2a $$\begin{aligned}&s({\varPhi } (A_1 A_2 )|{\varPhi } (A_1 A_1 ))\ge 0,\quad s({\varPhi } (A_2 A_3 )|{\varPhi } (A_2 A_2 ))\ge 0,\nonumber \\&s({\varPhi } (A_1 A_3 |{\varPhi } (A_3 A_3 )>0 \end{aligned}$$

or triples $\left( {(A_1 ,A_2 ),(B_1 ,B_2 ),(C_1 ,C_2 )} \right)$ such that

7.2b $$ \begin{aligned} \begin{array}{l} {\varPhi } (A_2 A_2 ,B_2 B_2 ,C_2 C_2 )={\varPhi } (A_1 A_1 ,B_1 B_1 ,C_1 C_1 )\ \& \\ s({\varPhi } (A_1 A_2 ,B_1 B_1 ,C_1 C_1 )|{\varPhi } (A_1 A_1 ,B_1 B_1 ,C_1 C_1 ))\ge 0, \\ s({\varPhi } (A_2 A_2 ,B_1 B_2 ,C_1 C_1 )|{\varPhi } (A_2 A_2 ,B_1 B_1 ,C_1 C_1 ))\ge 0,\\ s({\varPhi } (A_2 A_2 ,B_2 B_2 ,C_2 C_1 )|{\varPhi } (A_2 A_2 ,B_2 B_2 ,C_1 C_1 ))>0. \end{array} \end{aligned}$$

(7.1) together with the assumption that there are no unprotected dimorphisms makes that the resident population stays monomorphic so that there remains a close coupling between individual and allelic phenotypes. It thus looks that we are in business. However, there is still trouble to come.

At first sight a blanket assumption implying both (7.1) and (7.2) appears to be that $s(Y|X)=f(Y,X)\left( {\psi (Y)-\psi (X)} \right)$ and that mutational steps are constrained to be small, making the genotype-to-phenotype map close to additive (see e.g. Metz and Kovel 2013, p. 3). With additive genetics there even exists a counterpart to (2.2): A new mutant can be described by its additive contribution; if this contribution is zero its invasion fitness is zero. However, near the singularities of $\psi$ things go awry: if the mutational steps have length up to $\varepsilon$, then when the trait substitution sequence has come within a $2\varepsilon$ distance of a singularity a mutation may occur that leads to heterozygote superiority or inferiority (even when near ess-es the map from genotypes to phenotypes stays close to additive, as is generally the case, the map from phenotype to fitness, and hence the combined map from genotype to fitness becomes quadratic). This problem cannot be repaired in a biologically meaningful way: the only way to choose ${\varPhi }$ such that problems of this type are excluded is to assume a connection between the genotype-to-phenotype map and the ecology, with the effect of mutations becoming smaller the closer the current phenotype is to a singularity of $\psi$. So we have to be content with the fact that frequency independence as defined by (7.1) and (7.2) can only reign up to a close distance of the singularities of $\psi$. This actually suffices for most practical purposes as in nature ESSes are never realised in all mathematical strictness anyway. At best real populations form clouds of trait values close to theoretically calculated ESSes.

Ecologists rarely seem to bother with the possibility of heterozygote superiority due to their focus on how the interactions of individuals shape ESSes. Effects that hamper Mendelian evolution compared to its clonal counterpart, such as heterozygote superiority, are referred to as genetic constraints. ESSes calculated on the assumption that there are no genetic constraints are referred to as Ideal Free (IF). Under the IF assumption ESSes are determined by the ecology, independent of the mode of reproduction. Moreover, polymorphisms due to heterozygote superiority, at least those that go with a community dynamical equilibrium, can be resolved by mutants that produce the superior phenotype in all genetic backgrounds (However, as this requires a non-additive genotype-to-phenotype map, it does not resolve the genetic constraints that for smooth genotype-to-phenotype maps kick in during the final phase of the approach to an ESS as outlined in the previous paragraph).

*Terminology  Effective reproductive output:* reproductive output that contributes to the next generation, as opposed to reproductive output that goes wasted like sperm that fail to fertilise.

Finally, when everybody is born equal but for genetic differences and the community dynamics converges to an equilibrium (BE&CE), ESC-s equalise effective lifetime reproductive outputs of the phenotypes and do not depend on the reproductive mode (but may be more difficult to reach due to the constraints of the Mendelian mechanism on the population dynamics). Hence, under the IF plus BE&CE assumption we can use the concepts of frequency independence and weak frequency dependence as for clonal reproducers while also including ESCs in the argument (e.g., to conclude that protected polymorphic ESC-s do not exist), but this does not extend to the meso-evolutionary dynamics of which they are the equilibria, unless mutational steps are small and we decide not to worry about a little fuzz around a predicted ESS.

## Closing remarks

In this paper we argued for ecology-oriented concepts of frequency (in)dependence. Making a few reasonable assumptions we ended up with equating frequency independence with the existence of an optimisation principle. As “realistic” models with an optimisation principle are the exception rather than the rule, we also went for the next best thing, models having a weakly frequency dependent fitness function. For these models we attempted to elucidate to what extent they share relevant properties with models having frequency independent fitness functions. Of course the devil is in the meaning of “relevant”. For example, weakly frequency dependent fitness functions can differ qualitatively from frequency independent ones in that the frequency independent ones only allow neutral polymorphisms whereas the weakly frequency dependent ones may contain pockets of strong frequency dependence, i.e., engender some protected polymorphisms. However, the set of such polymorphisms is small and cannot have evolutionarily lasting members.

**Fig. 2:**
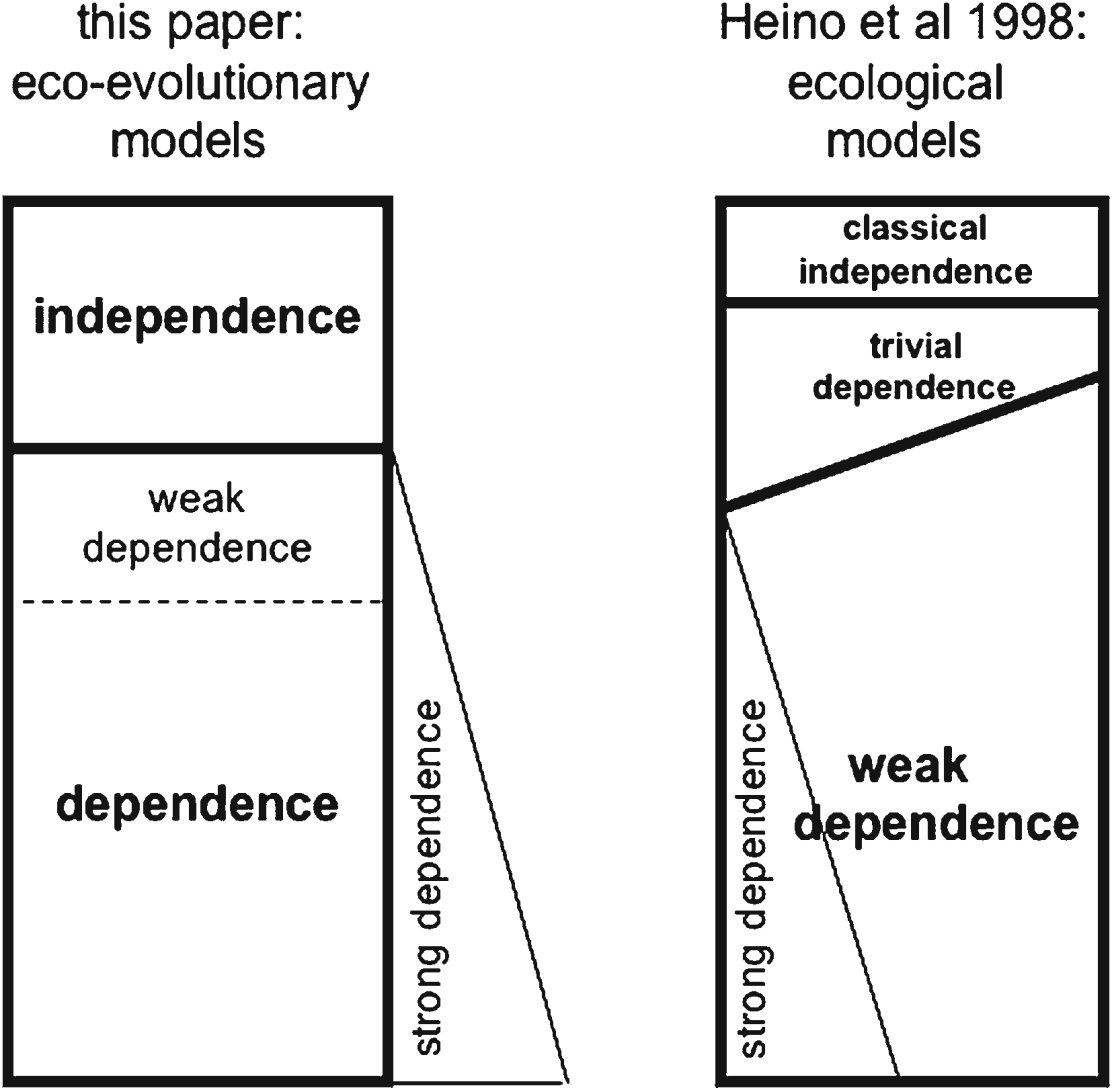
Inclusions between the different sorts of frequency dependence introduced in this paper and in Heino et al. (1998). The reason for drawing in the *left diagram* the strong frequency dependence region to the right of the main box is that this box refers to eco-evolutionary models, whereas strong frequency dependence refers to purely ecological models, as does the main box in the *right diagram*. The reason for drawing the border between trivial and weak frequency dependence tilted in the *right diagram* where the border between frequency independence and dependence in the *left diagram* is drawn horizontal is that when the definition of frequency independence underlying the *left diagram *is applied to single trait-*n*-tuples it does come close to but does not precisely equal the union of classical frequency independence and trivial frequency dependence in the *right diagram*

Figure 2 diagrammatically represents the relationships between the various concepts. For a biologist our weak frequency dependence may feel a little ambiguous. According to the definition it contains frequency independence, whereas the main usefulness of the term is, as the name suggests, in characterising a subset of frequency dependent fitness functions that by their nature generate an evolutionary dynamics qualitatively similar to that generated by frequency independent ones. However, this is no more than the usual discrepancy between the folk philosophical feeling about the proper delineation of concepts and the mathematician’s rule that concepts should be engineered to allow the tersest possible formulation of logically precise arguments.

The concepts compared in Fig. 2 all refer to invasion fitnesses, and hence to meso-evolution. Remains the question how they relate to the ancestral concept of frequency dependence from population genetics. The short answer is that they are incommensurable, with formula (2.3) forming the only point of contact between the two underlying universes of discourse. There also is a somewhat longer answer. The classical definition of frequency dependence, applicable to discrete time models without generation overlap, implies that the discrete time fitnesses of the different genotypes can be written as an environmentally dependent scalar factor times a positive scalar that depends only on the genotype. If we apply this restriction to invasion fitnesses of clonal models, this implies both (3.4) and (3.5), and hence these models have an optimisation principle, and thus are also frequency independent in a meso-evolutionary sense. However, the converse cannot hold good, for where population genetical fitnesses govern the fine detail of a gene substitution (thanks to the simplicity of the attendant ecological assumptions), invasion fitnesses only tell something about its beginning or end. Hence, any meso-evolutionary frequency independence concept is necessarily more relaxed than a micro-evolutionary counterpart. Moreover, in line with the focus of evolutionary ecologists on ESSes, we based our new concept of frequency independence only on the signs of the fitness function, thus making it even more relaxed. However, the two concepts coincide in that they lead to the same qualitative consequences for the properties of meso-evolutionary trajectories generated by frequency independent eco-evolutionary models. Finally, for Mendelian populations all comes loose if we leave sufficient room for the genotype-to-phenotype map’s potential for messing things up. Ways to deal with this are discussed in Sect. 7.

Ecologists relatively early on enriched population genetical terminology by in addition to the concept of frequency dependence—the ratios of fitnesses depend exclusively on the gene frequencies—introducing the concept of density dependent fitnesses—the ratios depend exclusively on the densities—(e.g., Roughgarden 1976), with the definition of the latter term also happily situated within the population genetical universe of discourse. Moreover, in arguments using this term it was assumed implicitly that fitnesses decrease with density (Or rather, we never have encountered a model in a population genetical book or paper where such was not the case). Moreover, rock-scissor-paper cycles are generally perceived as the result of frequency dependence. Together these two conditions, fitnesses that decrease with density and absence of rock-scissor-paper cycles, if true for an eco-evolutionary model, imply that it has an optimisation principle, evolution maximises minus the population density, hence those density dependent fitnesses are also frequency independent in a meso-evolutionary sense.

As final message we want to point at an interesting halfway philosophical problem. There exist various criteria by which it can be checked whether an invasion fitness function is frequency independent, or weakly frequency dependent. In particular we can look whether the associated PIPs have the required shape (Metz et al. 2008, Gyllenberg et al. 2011). Another matter is how to find for a given weakly frequency dependent fitness function an associated approximate optimisation principle $\psi$. Gyllenberg and Service (2011) give a construction for the frequency independent case. However, this construction requires infinitely many basic operations and to calculate $\psi$ recourse has to be taken to Monte Carlo methods. On the other hand, there are by now many cases in which optimisation principles have been found by some finitary construction coming from an inspired analysis of the eco-evolutionary model (for a systematic overview of one class of examples see Rueffler et al. 2013). Only such a priori constructed optimisation principles can act as computational tools, the inferred ones only help in categorising observations on model outcomes. It would be interesting to see this gap bridged by the development of tools that, given an eco-evolutionary model with weakly frequency dependent fitness function, hint at possible formulas for and/or interpretations of the inferred close by optimisation principle.

## Figures and Tables

**Fig. 3:**
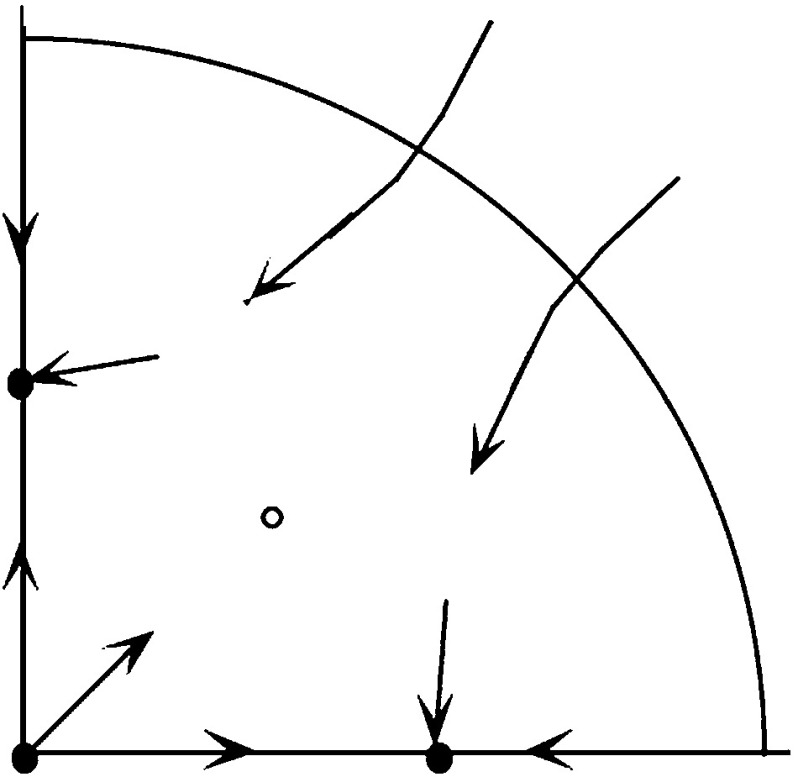
Diagram illustrating the main features of the community dynamics in the case that the evolving parameters, *Y*, *X*, satisfy $s(Y|X) < 0$ and $s(X|Y) < 0$

## Appendix 1: Proof that a one-dimensional environment generically implies a sign anti-symmetric fitness function

Before we start with the proof, a remark about the domain of $\rho (Y|\cdot ):E\mapsto s(Y|E)$ is in order. In Sect. 3 we restricted this domain to $\mathcal {E} = E_{\text {attr}} (\mathcal {C})$, in order to be able to formulate requirements on the action of *E* necessary and sufficient for the existence of an optimisation principle. For if we start from a model formulation that allows calculating $\rho$ for a domain $\mathcal {E}'$ larger than $\mathcal {E}$, as is usually the case, it does not matter how $\rho$ depends on *E* in $\mathcal {E}'\backslash \mathcal {E}$, and hence we have to remove $\mathcal {E}'\backslash \mathcal {E}$ from the consideration, just as we have to remove any components of *E* that fail to influence $\rho$ for any value of *Y*. However, necessary conditions for the existence of an optimisation principle were far from the mind of Heino et al. (1998), and below we follow their thinking and require *E* to act 1-dimensionally for any *E* for which *s* can be defined through a thought experiment where the environment is imposed instead of restricting to environments naturally generated by the attractors of the community dynamics.

Generically a fitness function is sign anti-symmetric iff there are no regions in $\mathcal {X} \times \mathcal {X}$ where $s(Y|X)>0$ and $s(X|Y)>0$ ($++$ regions) or regions where $s(Y|X)<0$ and $s(X|Y)<0$ ($--$ regions). Our goal is to prove by contradiction that the $++$ and $--$ regions are empty, at least generically.

As we are only after a proof of principle we make a number of additional assumptions, which can probably be relaxed quite a bit. First of all we assume that the dynamics of the population densities can be modelled using a system of ODEs (on the assumption that any population model worth its salt can be approximated uniformly (in the input from other members of the community) with such models). Secondly we assume that the environmental feedback is direct instead of mediated by other species in the community. Thirdly we assume that all considered morphs can invade the constant virgin environment, and have a unique single morph attractor, which is either an equilibrium or a limit cycle.

First we note that (2.2) applies not just for resident attractors but for any population trajectory where a population stays bounded and stays away from extinction, where we define *s* just as $$s(Y|E):=\mathop {\lim }\nolimits _{t\rightarrow \infty } \ln {\left( {n_Y (t)} \right) }/t$$, whenever the limit exists, $$n_Y =\mathbf{1}^{T}N_Y $$, $$N_Y $$ the population state of *Y*-morphs living in the environment *E*.

As a next step we observe that when $\rho (Y|E)=\beta \left( {Y|\varphi (E)} \right)$, $\varphi :\mathcal {E}'\rightarrow \mathbb {R}$, the existence of a solution to $\rho (X_1 |E)=0=\rho (X_2 |E)$ is equivalent to the existence of a solution of $\beta (X_1 |\varphi ^{*})=0=\beta (X_2 |\varphi ^{*})$, which is non-generic among functions $\beta :\mathcal {X}\times \mathbb {R}\rightarrow \mathbb {R}$. So generically $\rho (X_1 |E)=0=\rho (X_2 |E)$ does not have a solution.

A point $\{X_1 ,X_2 \}$ in a $++$ region would correspond to a protected dimorphism, requiring that $\beta \left( {X_2 |\varphi \left( {E_{\mathrm {attr}} (\{X_1 ,X_2 \})} \right) } \right) =\beta \left( {X_2 |\varphi \left( {E_{\mathrm {attr}} (\{X_1 ,X_2 \})} \right) } \right) =0$, which generically cannot be satisfied.

The idea for handling the $$--$$ regions is illustrated in Fig. 3. Denote the state space for a monomorphic case as $$\mathcal {N}$$, and the state space for the dimorphic case as $$\mathcal {M}:=\mathcal {N}\times \mathcal {N}$$. The two subsets of $$\mathcal {M}$$ corresponding to monomorphisms we denote as $$\mathcal {N}_1 :=\mathcal {N}\times \{\mathbf{0}\}$$ and $$\mathcal {N}_2 :=\{\mathbf{0}\}\times \mathcal {N}$$, and the virgin state as $$V\in \mathcal {N}_1 \cap \mathcal {N}_2 $$. By assumption $$\mathcal {N}_1 \backslash \{V\}$$ and $$\mathcal {N}_2 \backslash \{V\}$$ each contain a unique monomorphic attractor of the dimorphic dynamics. These attractors have an open domain of attraction the complement of which is forward invariant. Hence it contains at least one $$\omega $$-limit, to be denoted as $${\varOmega } $$. The intersections of $${\varOmega } $$ with $$\mathcal {N}_1 \backslash \{ V\}$$, $$\mathcal {N}_2 \backslash \{ V\}$$ and $$\{V\}$$ are necessarily empty, in the case of the former two since they are part of the domain of attraction of the monomorphic attractors, and of the latter since *V* is repelling. Since $${\varOmega } $$ is closed, both $$n_{X_1 } $$ and $$n_{X_2 } $$ stay bounded away from zero and by the general assumption that population densities stay bounded also from $$\infty $$. So once again $$\rho (X_i |E)$$, where *E* now is generated by the dynamics of $$(N_1 ,N_2 )$$ on $${\varOmega } $$, is well defined, and $$\rho (X_i |E)=0$$, so that the assumption that $$\rho (Y|E)=\beta \left( {Y|\varphi (E)} \right) $$ generically leads to a contradiction.

## Appendix 2: Algebraic relations for frequency independent fitness functions

A $\hbox {C}^{2}$ fitness function $s:\mathcal {X}\times \mathcal {X}\rightarrow \mathbb {R}: \quad (Y,X)\mapsto s(Y|X)$ is frequency independent iff there exist $\hbox {C}^{2}$ functions $\psi :\mathcal {X}\rightarrow \mathbb {R}$ and $f:\mathcal {X}\times \mathcal {X}\rightarrow \mathbb {R}^{+}$ such that

A.1 $$\begin{aligned} s(Y|X)=f(Y,X)\left( {\psi (Y)-\psi (X)} \right) . \end{aligned}$$

Expanding around the diagonal gives

A.2 $$\begin{aligned}&f(X+V,X+U)\left( {\psi (X+V)-\psi (X+U)} \right) \nonumber \\&\quad =f(X,X)\partial \psi (X)(V-U) \nonumber \\&\qquad +\,\frac{1}{2}V^{T}\left( {\partial ^{T}\psi (X)\partial _1 f(X,X)+\partial _1^T f(X,X)\partial \psi (X)+f(X,X)\partial ^{T}\partial \psi (X)} \right) V\nonumber \\&\qquad +\,\frac{1}{2}V^{T}\left( {\partial ^{T}\psi (X)\partial _2 f(X,X)-\partial _1^T f(X,X)\partial \psi (X)} \right) U \nonumber \\&\qquad +\,\frac{1}{2}U^{T}\left( {-\partial ^{T}\psi (X)\partial _1 f(X,X)+\partial _2^T f(X,X)\partial \psi (X)} \right) V \nonumber \\&\qquad +\,\frac{1}{2}U^{T}\left( {-\partial ^{T}\psi (X)\partial _2 f(X,X)-\partial _2^T f(X,X)\partial \psi (X)-f(X,X)\partial ^{T}\partial \psi (X)} \right) U \nonumber \\&\qquad +\,\hbox {h.o.t.} \end{aligned}$$

Hence

A.3 $$\begin{aligned} B(X)= & {} f(X,X)\partial \psi (X),\end{aligned}$$

A.4 $$\begin{aligned} 2\mathbf{C}_{00} (X)= & {} \partial ^{T}\psi (X)\partial _1 f(X,X)+\partial _1^T f(X,X)\partial \psi (X)+f(X,X)\partial ^{T}\partial \psi (X), \nonumber \\ 2\mathbf{C}_{01} (X)= & {} \partial ^{T}\psi (X)\partial _2 f(X,X)-\partial _1^T f(X,X)\partial \psi (X), \nonumber \\ 2\mathbf{C}_{10} (X)= & {} -\partial ^{T}\psi (X)\partial _1 f(X,X)+\partial _2^T f(X,X)\partial \psi (X), \nonumber \\ 2\mathbf{C}_{11} (X)= & {} -\partial ^{T}\psi (X)\partial _2 f(X,X)-\partial _2^T f(X,X)\partial \psi (X)-f(X,X)\partial ^{T}\partial \psi (X). \nonumber \\ \end{aligned}$$

(5.5) follows from (A.3) and $G(X)=B^{T}(X)$. (5.6) follows from (A.4) and the fact that at ess-es $G(X^{*})=0$ and hence $\partial \psi (X^{*})=0$.

To conclude from (5.5) that for the CE $\psi$ increases over evolutionary time, provided $\varvec{\Sigma }$ is non-degenerate and thus positive definite and $G(X)\ne 0$, $\left\langle {\partial \psi (X),{\Sigma } G(X)} \right\rangle =c(X)\partial \psi (X){\Sigma } \partial ^{T}\psi (X)>0$, with $c(X)={\left\| {G(X)} \right\| }/{\left\| {\partial \psi (X)} \right\| }$. Hence the vector field of the CE except when $G(X)=0$ everywhere points in a direction in which $\psi$ increases.

## Appendix 3: Proof of Lemma 6.3

The intuition behind the proof is as follows: Let $C^{*}=\{X_1^*,\ldots ,X_n^*\}$ be a hypothetical esc of *s*. For $\tilde{s}$ the values of $\psi (\tilde{X})$, $\tilde{X}\in \tilde{C}\in \tilde{\mathcal {C}}$, $\tilde{\mathcal {C}}$ the polymorphisms associated with $\tilde{s}$, are equal. Since *s* is a slight perturbation of $\tilde{s}$, $\psi (X_i^*)-\psi (X_j^*)=O(\varepsilon )$. When $X_i^*$ and $X_j^*$ are not close, they should correspond to different singular points of $s:Y\mapsto s\left( {Y|\{X_1^*,\ldots ,X_n^*\}} \right)$, and hence be close to different singular points of $\psi$. By making $\varepsilon$ small enough we get a contradiction. The case where $X_i^*$ and $X_j^*$ are close is considered further below.

The problem with this argument is that $\tilde{s}$ is not defined for $\{X_1 ,\ldots ,X_n \}$ when some $\psi (X_i )\ne \psi (X_j )$, so that the meaning of the phrase “*s* is a slight perturbation of $\tilde{s}$” does not have the usual meaning. Hence we have to proceed a little differently. We only consider dimorphisms. The case for higher polymorphism is similar but needs more verbiage.

We again consider the case where $$X_1^*$$ and $$X_2^*$$ are appreciably different. Due to the narrowness of the coexistence region there exists an $$\varepsilon _1 =O(\varepsilon )$$ and points $${C}'=\left\{ {X_1^*,X_2 } \right\} $$ on the boundary of $$C_2 $$ where $$X_1^*$$ has zero density and $${C}''=\left\{ {X_1 ,X_2^*} \right\} $$ on the boundary where $$X_2^*$$ has zero density with $$\left\| {X_i -X_i^*} \right\| <\varepsilon _1 $$. In these points

A.5 $$\begin{aligned} s(Y|{C}')=s(Y|X_i )=f(Y,X_i )\left( {\psi (Y)-\psi (X_i )} \right) +O(\varepsilon ) \end{aligned}$$

and

A.6 $$\begin{aligned} \partial _1 s(X_i |{C}')=\partial _1 s(X_i |X_i )=f(X_{i},X_{i})\partial \psi (X_i )+O(\varepsilon ). \end{aligned}$$

Hence $X_1^*$ and $X_2^*$ lie within a $\delta$-distance of two different singular points of $\psi$, to be called $\tilde{X}_1^*$ and $\tilde{X}_2^*$, where we can make $\delta$ as small as we want by an appropriate choice of $\varepsilon$. The regularity of $\psi$ guarantees that there exists a $\delta _1$ such that the values of $\psi$ at its singular points are at least $\delta _1$ apart. The $\hbox {C}^{2}$ smoothness of $\psi$ means that by choosing $\varepsilon$ small enough we can make both $X_i^*-\tilde{X}_i^*$ sufficiently small to get $\psi (X_i^*)$ within a $\frac{1}{2}\delta _1$-distance of $\psi (\tilde{X}_i^*)$, providing us with the sought after contradiction.

The case where $X_1^*$ and $X_2^*$ are close is covered by a look at Fig. 1, and using (i) that an esc stays an esc when we restrict ourselves to a 1-dimensional manifold in *X*, which we can choose so as to pass through $X_1^*$ and $X_2^*$, and (ii) that the case for higher polymorphisms can be reduced to that for dimorphisms by forbidding the remaining phenotypes to mutate. [Here we implicitly use that the Hessians at the singular points of $\psi$ are non-singular as near points where the Hessian is singular the bifurcation away from the frequency independent case can be different from the pattern indicated in Fig. 1, with the boundaries of the coexistence region having a curvature that becomes higher and higher the closer one comes to the frequency independent case, so that it cannot be removed by blowing up the area around the singular point, as can be inferred from the middle panel in the third row of Figure 2 of Metz et al. (2008).]

## Footnotes

1 This convention also makes the average lifetime reproductive output in a stationary population equal to one, independent of reproduction type or structure.

2 These authors also give a sample from the literature of attempts to capture the concept as intuited in the community, illustrating its vagueness.

3 e.g. Wright (1949), Fisher (1958), Lewontin (1958), Wright (1969); most other papers on frequency dependent models consider specific ecology derived issues (mainly evolutionary games).

4 A property is called robust if it stays intact under small perturbations of the model.
